# Breast Cancer Brain Metastases: Implementation and Characterization of a Mouse Model Relying on Malignant Cells Inoculation in the Carotid Artery

**DOI:** 10.3390/cells12162076

**Published:** 2023-08-16

**Authors:** Joana Godinho-Pereira, Daniela Vaz, Inês Figueira, Joana Aniceto-Romão, Istvan Krizbai, Rui Malhó, João Rocha, Manuela Colla Carvalheiro, Sandra Simões, Maria Manuela Gaspar, Maria Alexandra Brito

**Affiliations:** 1 iMed.ULisboa—Research Institute for Medicines, Faculty of Pharmacy, Universidade de Lisboa, Av. Prof. Gama Pinto, 1649-003 Lisbon, Portugal; joanagpereira@ff.ulisboa.pt (J.G.-P.); danielaa.vaz22@gmail.com (D.V.); ines.figueira88@gmail.com (I.F.); joana.romao@live.com.pt (J.A.-R.); jrocha@ff.ulisboa.pt (J.R.); manuela.colla@campus.ul.pt (M.C.C.); ssimoes@ff.ulisboa.pt (S.S.); mgaspar@ff.ulisboa.pt (M.M.G.); 2 Department of Pharmaceutical Sciences and Medicines, Faculty of Pharmacy, Universidade de Lisboa, Av. Prof. Gama Pinto, 1649-003 Lisbon, Portugal; 3 Farm-ID—Faculty of Pharmacy Research and Development Association, Av. Prof. Gama Pinto, 1649-003 Lisbon, Portugal; 4 Institute of Biophysics, Biological Research Centre, Eötvös Loránd Research Network (ELKH), 6726 Szeged, Hungary; krizbai.istvan@brc.hu; 5 Institute of Life Sciences, Vasile Goldis Western University of Arad, Str. Liviu Rebreanu 86, 310414 Arad, Romania; 6 BioISI—Biosystems and Integrative Sciences Institute, Faculty of Sciences, Universidade de Lisboa, Campo Grande, 1746-016 Lisbon, Portugal; r.malho@fc.ul.pt; 7 Department of Pharmacy, Pharmacology and Health Technologies, Faculty of Pharmacy, Universidade de Lisboa, Av. Prof. Gama Pinto, 1649-003 Lisbon, Portugal

**Keywords:** blood–brain barrier, brain metastases, breast cancer, Ki-67, intracarotid injection, mouse model, cytokeratin, peripheral metastases, proliferation, tomato lectin

## Abstract

Breast cancer (BC) brain metastases (BCBM) is a severe condition frequently occurring in the triple-negative subtype. The study of BCBM pathogenesis and treatment has been hampered by the difficulty in establishing a reliable animal model that faithfully recapitulates the preferential formation of brain metastases. The injection of BC cells in the carotid artery of mice has been proposed but the procedure is challenging, with the metastatic pattern being scarcely characterized. In this work, we thoroughly describe an improved procedure, highlighting the tricks and challenges of the process, and providing a characterization of the brain and peripheral metastatic pattern at the cellular and molecular level. Triple-negative BC (4T1) cells were inoculated in the common carotid artery of BALB/c mice. Brains and peripheral organs were harvested at 7–14 days for the histological characterization of the metastases’ pattern and the immunofluorescence analysis of specific markers. With our surgical procedure, both mouse death and procedure-associated weight loss were negligible. Brain metastases mostly occurred in the hippocampus, while sparse peripheral lesions were only detected in the lungs. Brain-colonizing BC cells presented proliferative (Ki-67) and epithelial (pan-cytokeratin and tomato lectin) features, which account for metastases’ establishment. The presented surgical approach constitutes an important and reliable tool for BCBM studies.

## 1. Introduction

Breast cancer (BC) is the most diagnosed and leading cancer in women, with 2.3 million new cases estimated in 2020 [1]. With the increased BC patients’ survival due to the remarkable improvements in early detection and therapies for primary tumour treatment [2], distant organ metastases arise as the foremost concern for BC survivors. In fact, 20–30% of them die of metastatic disease [3], with a less than 30% 5-year survival rate, even with adjuvant chemotherapy [4]. Approximately 15% of patients with BC develop brain metastases (BM) [5], a devastating problem with a survival rate of only 20% 1 year after the diagnosis [6], particularly for the triple-negative (TN) BC (TNBC) subtype [7,8].

Although lymphovascular dissemination is associated with an increased risk of distant metastases and a poor survival [9,10], breast to brain metastasization follows a vascular distribution mainly through a haematogenous spread [6]. However, the current understanding of the pathogenesis of breast cancer brain metastases (BCBM) and the assessment of new therapeutics has been hampered by the difficulty in the establishment of appropriate animal models that faithfully recapitulate the formation of BC metastases preferentially in the brain with the haematogenous route. Several non-mammalian organisms have been used to study the metastasization process in vivo due to the simplicity and ease of use, but they fail to recapitulate the complex processes associated with the metastatic cascade in mammalians. Therefore, mammalian organisms, particularly mice, have become the most widely used [11]. Mouse models rely on the transplantation of tumours, organoids or tumour cells from humans (xenograft models) or mice (syngeneic models). Xenograft models require the use of immunocompromised mice to avoid the rejection of human cells, but have the advantage of using a patient’s tumour tissue [12], which is relevant for personalized medicines. Syngeneic models rely on the implantation into immunocompetent mice of the same background, retaining an intact immune system, which is particularly relevant for studies of immunity and immunotherapy [13].

To promote BM formation, cells can be inoculated orthotopically (in the organ they originated from) [11] or ectopically (in a different place from the organ they originated from) [11,14]. Orthotopic injection routes, such as the cell injection in the mammary fat pad of mice, have the benefit of recapitulating all the metastatic processes. This method can be used for primary tumour models, as well as for spontaneous metastatic ones [15]. Regarding BM, the fat-pad injection model has the hurdle of spreading to several metastatic locations, and not only to the brain [15]. Ectopic inoculation may rely on different injection routes, such as tail vein, intracardiac or intracarotid [11,14]. The majority of metastatic tumour models rely on intravenous injection to deliver tumour cells [16]. Even though this injection route presents a very high survival rate and does not require great microsurgical skills, the intravenous injection in the tail vein facilitates the lodging of cells in peripheral organs such as the lungs, as they are the first encountered organ with a considerable capillary bed [17]. This may lead to death by large numbers of lung metastases before the BCBM are developed in these models [18]. Another ectopic model consists of the intracardiac injection, which is useful to determine tumour cell predisposition for specific secondary environments [16]. However, it presents disadvantages, including the formation of tumours within the heart and the direction of the tumour cell inoculum to sites other than the brain, adding to a decreased reproducibility resulting from the blind nature of the inoculation and a fairly high degree of procedural mortality [19]. Alternative injection routes primarily targeting the brain, such as direct intracerebral, arose to induce experimental BCBM through the puncturing of the mice’s skull [20,21]. Despite its utility in leptomeningeal melanoma metastases research [22], direct intracerebral injection is most promising in primary local growth models, such as glioblastoma [23], rather than metastatic ones, as it can only replicate the final stage of the metastatic process [14]. Importantly, the impact of the administration route on therapeutics’ uptake [24], where the intracranial injection may induce alterations on the blood–brain barrier (BBB) and blood–tumour barrier, has been demonstrated, increasing the brain penetration of non-targeted pharmaceuticals associated with the injection method, which does not occur in intracardiac and tail intravenous injection routes [24].

Although in vivo models have been put into place to effectively deliver cells into the brain [23] and promote preferential BM formation [16,20], due to the nature of certain procedures, these models may lead to unfortunate outcomes prior to BM development. Therefore, the intracarotid injection represents a preferential highway to the brain, allowing cancer cells’ efficient delivery to this site. Moreover, it allows for replicating the circulation step as well as both cell extravasation and brain colonization processes. On top of all the aforementioned advantages [19], the intracarotid injection model provides a unique platform for the study of BCBM or other BM development processes in vivo, since it allows for the study of BM pathogenesis and processes, immune responses, biomarkers and potential therapeutic target discovery.

In this work, we aimed to employ and minutely characterize a robust intracarotid animal model of BCBM development that closely reproduces the clinical setting of the process, describing in depth the tricks and challenges of the procedure. We characterized the formation and development of metastases over time in the brain and peripheral organs, and studied the metastatic properties of BC cells (BCCs) during colonization and metastases’ establishment. With our in vivo model, mouse death was negligible, and metastases occurred preferentially in the brain. An analysis of the metastatic properties of BCCs upon colonization showcased an enhancement of the proliferative capacity and the acquisition of an epithelial phenotype accompanied by a stabilization of the metastases’ number in the brain later in time, reflecting the passage from the extravasation process to brain colonization. All in all, the implementation of such an animal model, as well as its characterization, contributes to an increase in the knowledge of BCBM development and constitutes a reliable tool to investigate the potential of therapeutic strategies directed at such pathology.

## 2. Materials and Methods

### 2.1. Cell-Culture Conditions

The murine mammary carcinoma TNBC 4T1 cell line (ATCC, Middlesex, UK) was used. The 4T1 cells were cultured in RPMI 1640 (Sigma Aldrich, St. Louis, MO, USA) and supplemented with 2 mM of L-glutamine (Biochrom AG, Berlin, Germany), a 1% antibiotic–antimycotic solution (Sigma Aldrich) and a 5% (*v*/*v*) heat-inactivated foetal bovine serum (FBS, Sigma Aldrich). Cells were maintained at 37 °C in a humid atmosphere enriched with 5% CO_2_.

### 2.2. Mouse Model of BCBM

#### 2.2.1. Animals

Female BALB/c mice, 8–10 weeks old, were obtained from the Gulbenkian Institute of Science (Oeiras, Portugal). Animals were kept under standard hygiene conditions, fed commercial chow and given acidified drinking water ad libitum. All animal experiments were conducted according to the animal welfare organization of the Faculty of Pharmacy, University of Lisbon, approved by the competent national authority Direção Geral de Alimentação e Veterinária (DGAV license, 003866; 10 March 2023) and conducted in accordance with the EU Directive (2010/63/UE) and Portuguese laws (DR 113/2013, 2880/2015, 260/2016 and 1/2019) for the use and care of animals in research.

#### 2.2.2. Procedure

**Figure 1:**
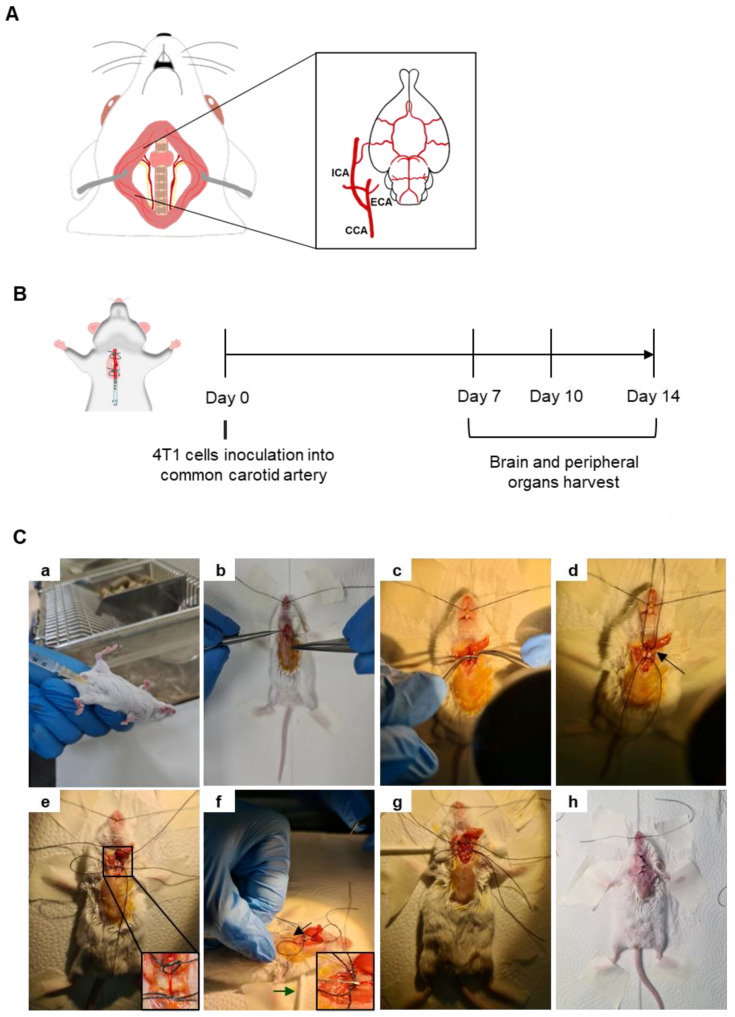
Mouse model of breast cancer brain metastases (BCBM) induced with intracarotid injection of malignant cells. Schematic representation of the common carotid artery (CCA), located laterally to the trachea, and ramified into the external carotid artery (ECA) and the internal carotid artery (ICA), which is essential for brain irrigation (**A**). Representation of the experimental design relying on the inoculation of breast cancer cells (4T1) into the CCA of BALB/c mice; sacrificing and organs harvesting took place 7, 10 and 14 days after cells inoculation (**B**). Representative images of the experimental procedure (**C**). Mice were anaesthetized with intraperitoneal (i.p.) injection of ketamine/medetomidine cocktail using a 25 G needle (**a**). After fur removal and skin disinfection, a small incision was made in the skin, and dissection of the surrounding tissue was performed to reach the CCA (**b**). The CCA was exposed and separated from the vagus nerve (**c**). Two suture lines were disposed below the CCA, a proximal and a distal one with loose knots, along with a cotton ball embedded in phosphate-buffered saline placed underneath the artery (arrow) (**d**). The proximal knot was tightened to block the blood flow into the point of incision (**e**). A cotton swab was positioned under the animal’s neck to provide better visibility of the surgical area (green arrow) and the administration of 4T1 cells was achieved using a 30 G needle (black arrow and inset) (**f**). After the injection of the cells, a tight knot was made in the distal line before the needle was carefully removed (**g**). The animal was sutured (**h**), placed into a heating pad until recovering conscientiousness and then moved into normal housing.

A mouse model of BCBM relying on the inoculation of 4T1 cells in the common carotid artery (CCA) (Figure 1A) of BALB/c mice, to induce preferential BM formation, was implemented and characterized based on an analysis of the metastatic pattern in the brain and peripheral organs 7, 10 and 14 days after 4T1 cell inoculation (Figure 1B). Mice were anaesthetized with an intraperitoneal (i.p.) injection of ketamine (75 mg/kg) and medetomidine (1 mg/kg) using a 25 G needle (B. Braun, Melsungen, Germany) (Figure 1Ca). After fur removal, the animal’s skin was disinfected using ethanol and povidone iodine (betadine^®^, Viatris, PA, USA). A 15–20 mm incision was performed in the skin using a number 11 scalpel blade (B. Braun) (Figure 1Cb) and a dissection of the surrounding tissue was conducted to expose and separate the right CCA from the vagus nerve (Figure 1Cc). To better expose the carotid, a cotton ball embedded in sterile phosphate-buffered saline (PBS) was placed underneath the artery, along with two silk sutures 3/0 (B. Braun) disposed underneath the artery (a proximal and a distal one with loose knots) (Figure 1Cd). The proximal knot was tightened to block the blood flow into the point of the incision (Figure 1Ce). A cotton swab was positioned under the animal’s neck to provide better visibility of the surgical area and 0.2 × 10^6^ 4T1 cells in 150 µL of PBS were inoculated in the right CCA using a 30 G needle (B. Braun) (Figure 1Cf). After the injection of the cells, a tight knot was created in the distal line before the needle was carefully removed (Figure 1Cg), and a haemostatic tampon (Nexcare^TM^, 3M, Spain) was placed onto the site of injection. The animal was sutured using single-interrupted sutures with a 2/0 silk thread (B. Braun) (Figure 1Ch), placed into a heating pad until recovering conscientiousness and then moved into normal housing. Animals’ weight was monitored daily, as an overall health welfare factor. A few food pellets were placed at the bottom of cages to facilitate food intake. To avoid pain, codeine (30 mg/500 mL) was added to drinking water ad libitum, on the day of the surgery and at postoperative days 1 and 2.

### 2.3. Organ Harvesting and Processing

Brains and peripheral organs were collected for a histological, fluorescence or immunofluorescence (IF) analysis. Anaesthetized mice were intracardially perfused with 20 mL of PBS, followed by 10 mL of a 4% (*w*/*v*) paraformaldehyde (PFA, Sigma Aldrich) solution to fix the tissues. The organs were harvested and postfixed overnight in 4% PFA at 4 °C and kept in PBS also at 4 °C until processing. The brains, lungs, kidneys and livers were paraffin embedded and serially cut into 4-μm-thick sections. To obtain different regions of the brain, coronal sections were created following the Bregma coordinates, namely the cerebellum, −6.12 mm, and cranial hippocampus, −1.82 mm. The processing and sectioning of samples were performed at the Histology and Comparative Pathology Laboratory at the Institute of Molecular Medicine João Lobo Antunes, Lisbon, Portugal.

#### 2.3.1. Histological Analysis

To depict metastases, a histological analysis of the brain and peripheral organs was performed through the haematoxylin-eosin (HE) staining of the sections at the Histology and Comparative Pathology Laboratory.

#### 2.3.2. Fluorescence and Immunofluorescence

Brain sections were processed for a fluorescence analysis of fluorescein-labelled tomato lectin (T-lectin, #FL-1171, Vector laboratories, Newark, CA, USA) or IF analysis of cytokeratin and Ki-67 (using the antibodies summarized in Table 1). Sections were deparaffinized in xylene and rehydrated through successive immersion in 100%, 96% and 70% ethanol, and finally tap water. Heat-mediated antigen retrieval was performed with 10 mM of sodium citrate, pH 6.0, for 15 min in a microwave. A permeabilization step was performed with 0.5% Triton X-100 (VWR International, Radnor, PA, USA) for 15 min, and tissue sections were blocked with 3% bovine serum albumin (BSA, Sigma Aldrich) containing 0.5% Triton X-100 for 60 min. To visualize BCCs with an epithelial phenotype, sections were incubated with T-lectin (1:500) or subjected to an IF analysis of pan-cytokeratin (Pancyt), whereas for the evaluation of BCC proliferation, Ki-67 immunostaining was undertaken. For the IF analysis, cells were firstly incubated with primary antibodies overnight at 4 °C and, secondly, with the respective fluorescent-labelled secondary antibodies, for 60 min at room temperature, in the dark, with antibodies diluted in a blocking solution. Between the several steps following the antigen retrieval treatment, the sections were washed 3× with PBS. Nuclei were labelled with Hoechst dye 33342 (20 µM, Thermo Fisher Scientific, Waltham, MA, USA) for 10 min, followed by mounting with SlowFade^®^ Diamond Antifade Mountant (Thermo Fisher Scientific). Negative controls with the omission of primary antibodies were used to exclude nonspecific binding or cross-reactivity.

### 2.4. Image Acquisition

Images of HE staining were obtained using an Olympus BX51 Microscope (Tokyo, Japan) equipped with a DFK 23U digital camera and Olympus Plan Apo 10× and 20× objectives. Fluorescence images were acquired using a fluorescence microscope (Olympus BX60) equipped with the appropriate filters (U-MNIB, U-MNG) and a Hamamatsu Orca R2 CCD camera.

### 2.5. Data Analysis

For the analysis of the metastases’ number and area, ImageJ 1.29xsoftware (National Institutes of Health, Bethesda, MD, USA) was used. For the BM number, the cranial hippocampus and cerebellum sections were analysed. As for the metastases’ area in the cranial hippocampus and cerebellum sections along with peripheral organs, the total tumour area was determined with the delimitation of each metastasis in the respective sections at each timepoint, and the results were expressed as the tumour area (mm^2^) for the coronal sections of the cranial hippocampus and cerebellum and as the tumour area per tissue area (mm^2^/mm^2^) for peripheral organs. The IF analysis relied on the examination of cranial hippocampus sections, a region prone to metastasis development [25]. In this sense, ten fields of the cranial hippocampus of each animal were acquired under the same conditions and analysed using the ImageJ 1.29x software and Icy 2.4.0.0 software (Institute Pasteur and France BioImaging, Paris, France).

### 2.6. Statistical Analysis

Results were analysed using GraphPad Prism^®^ 8.0 (GraphPad Software, La Jolla, CA, USA) and are expressed as the mean ± SEM. Data normality was tested with D’Agostino Pearson and Shapiro–Wilk tests. When normality was verified, the significance of the data difference was tested with a Student’s *t*-test and an analysis of variance with a one-way ANOVA test amongst different timepoints and organs. Multiple comparisons were performed with Tukey’s post-doc test. When no normality between data was observed, the significance of differences was evaluated with a Kolmogorov–Smirnov test (non-parametric). Differences were considered statistically significant when *p* < 0.05.

## 3. Results

### 3.1. Well-Established Metastases Are Detected in the Brain from 7 Days Onwards

**Figure 2:**
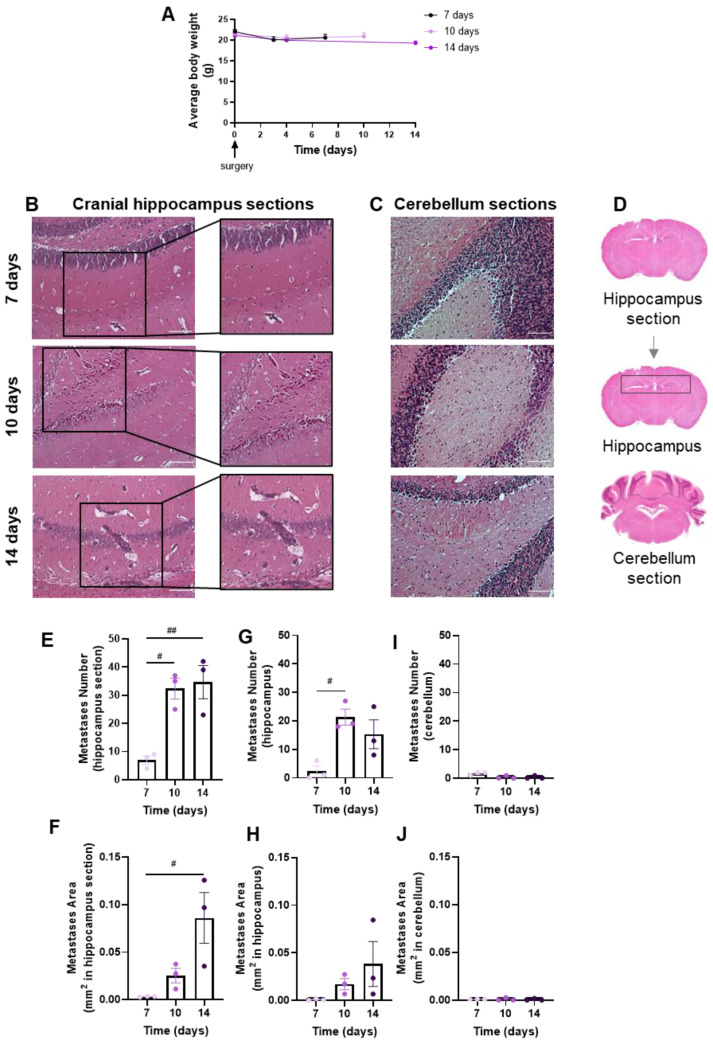
Profile of body weight and breast cancer brain metastases (BCBM) in the implemented mouse model. 4T1 cells were inoculated in the common carotid artery of BALB/c mice. Mice’s weight was evaluated to monitor the animals’ well-being throughout time until sacrifice (0 to 14 days), revealing a recovery of the slight surgery-associated weight loss (**A**). The pattern of BCBM formation was assessed with haematoxylin-eosin staining at different timepoints (7, 10 and 14 days). Images were taken with a 20× objective for both cranial hippocampus and cerebellum. Analysis of the cranial hippocampus sections revealed a time-dependent formation of metastases (insets) (**B**), while metastases were hardly detected in the cerebellum (**C**). Scale bar: 100 µm. Representative images of the analysed regions, namely coronal cranial hippocampus section, hippocampus region and cerebellum section (**D**). Quantification of metastases’ number (**E**) and area (**F**) in cranial hippocampus sections disclosed an increment throughout time. Quantification of metastases’ number (**G**) and area (**H**) in the hippocampus region revealed a significant increment throughout time. The semi-quantitative analysis of tumour number (**I**) and area (**J**) in the cerebellum revealed no significant presence of metastases. The results are expressed as mean ± SEM (*n* = 3 for each timepoint). A one-way ANOVA was used to assess the significant changes in parameters between indicated timepoints. For the average body weight, no significant changes were observed. Statistical significances are denoted as # *p* < 0.05 and ## *p* < 0.01 between timepoints.

Brain metastases’ development and evolution were characterized at different timepoints (7, 10 and 14 days) after the inoculation of 4T1 cells in the CCA of BALB/c mice (Figure 2). The observation and data collection of animals’ weight during the experiment time is of importance to ascertain the animals’ well-being. We observed that after surgery, all animal groups revealed a slight weight loss, though not statistically significant, which mostly recovered with time (Figure 2A). Visual inspection during the first hours after the surgery and daily until the end of the study did not reveal any neurologic symptoms (circling, rolling, hyperexcitability, convulsion, tremors, weakness of hind limbs and paralysis) in mice. No signs of suture site inflammation were observed.

The extension of brain metastases was assessed with the HE staining of coronal sections of the cranial hippocampus and cerebellum, as, in the original model, these were the most and the least affected brain regions, respectively [25]. Well-established metastases were observed at 7 days, increasing thereafter in cranial hippocampus sections (Figure 2B), whereas lesions were scarcely detected in cerebellum sections (Figure 2C). To assess metastases’ extension, a quantitative analysis was performed in cranial hippocampus sections, the hippocampus region and cerebellum sections (Figure 2D). The inspection of the cranial hippocampus sections for the metastases’ number (Figure 2E) and area (Figure 2F) revealed a significant increment throughout time. Additionally, a significant increase in the metastases’ number from 7 to 10 days post-injection was observed (Figure 2G), and an enlargement in the metastases’ area (Figure 2H) throughout time in the hippocampus region. In line with the qualitative observations, no changes were observed in the tumour number (Figure 2I) and area (Figure 2J) in the cerebellum sections.

### 3.2. Peripheral Breast Cancer Metastases Are Only Detected in the Lungs

**Figure 3:**
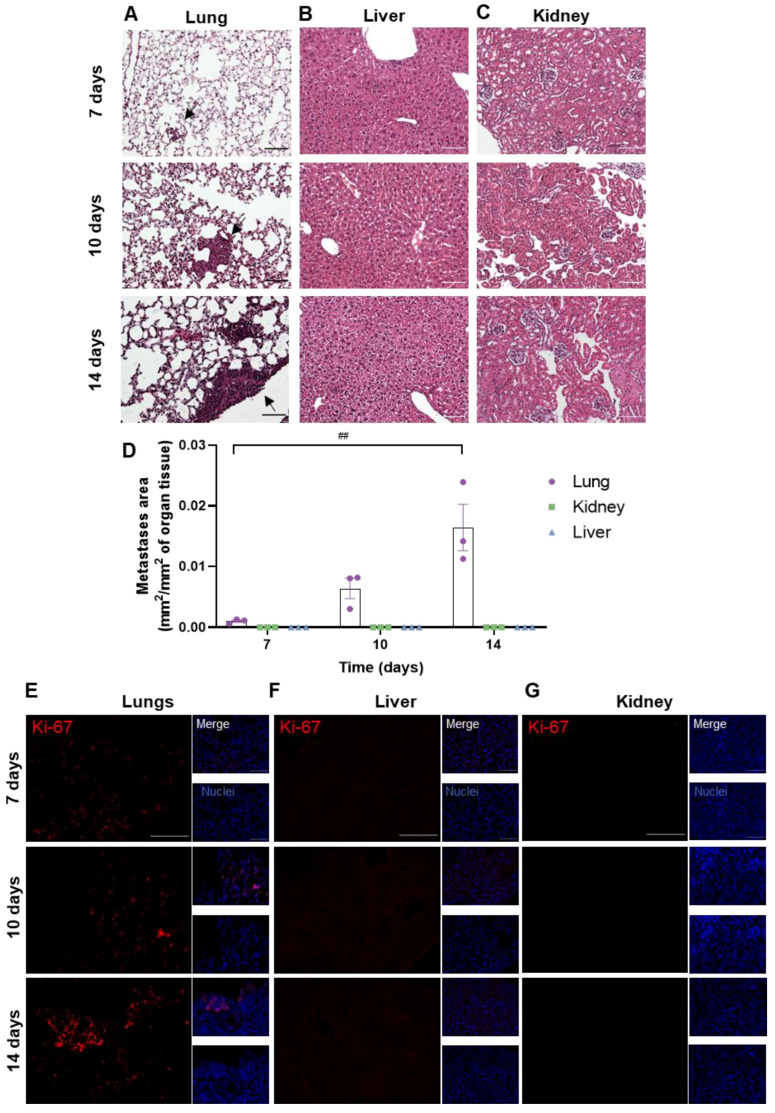
Profile of breast cancer metastases in peripheral organs. 4T1 cells were injected in the common carotid artery of BALB/c mice, and the presence of metastases was assessed with haematoxylin-eosin staining in lungs (**A**), liver (**B**) and kidneys (**C**) at different timepoints (7, 10 and 14 days). Scale bar: 100 µm. Fully developed metastases were observed from 7 days onwards in the mice’s lungs (black arrows), whereas no metastases were detected in the other peripheral organs. Observations were validated with the metastases’ area quantification (**D**). Immunostaining for Ki-67 (red), a proliferative marker, was assessed in lungs (**E**), liver (**F**) and kidneys (**G**) at different timepoints (7, 10 and 14 days). Scale bar: 100 µm. Hoechst dye 33342 was used as counterstaining for nuclei (blue). The results are expressed as mean ± SEM (*n* = 3 for each timepoint). A one-way ANOVA was used to determine significant changes in parameters between the different timepoints. Statistical significance is denoted as ## *p* < 0.01 between timepoints.

To assess whether metastases developed in peripheral organs, the lungs, kidneys and livers were harvested at different timepoints (7, 10 and 14 days) after the inoculation of 4T1 cells in the CCA. The presence of metastases was evaluated based on HE staining (Figure 3). Metastases were observed from 7 days onwards in the lungs (Figure 3A), whereas no metastases were detected in the other peripheral organs (liver and kidneys, Figure 3B,C). The metastases’ area was quantified and normalized for the analysed organ tissue area, showing an increase in tumour area throughout time in the lungs (Figure 3D). To corroborate the results obtained with HE, the Ki-67 staining of peripheral organs was performed. An increase in Ki-67-positive cells was observed from 7 days onwards in the lung parenchyma (Figure 3E), while no positive cells were found in the liver and kidneys (Figure 3F,G).

### 3.3. BCCs Acquire a Proliferative and Epithelial Phenotype during Brain Metastases’ Formation

**Figure 4:**
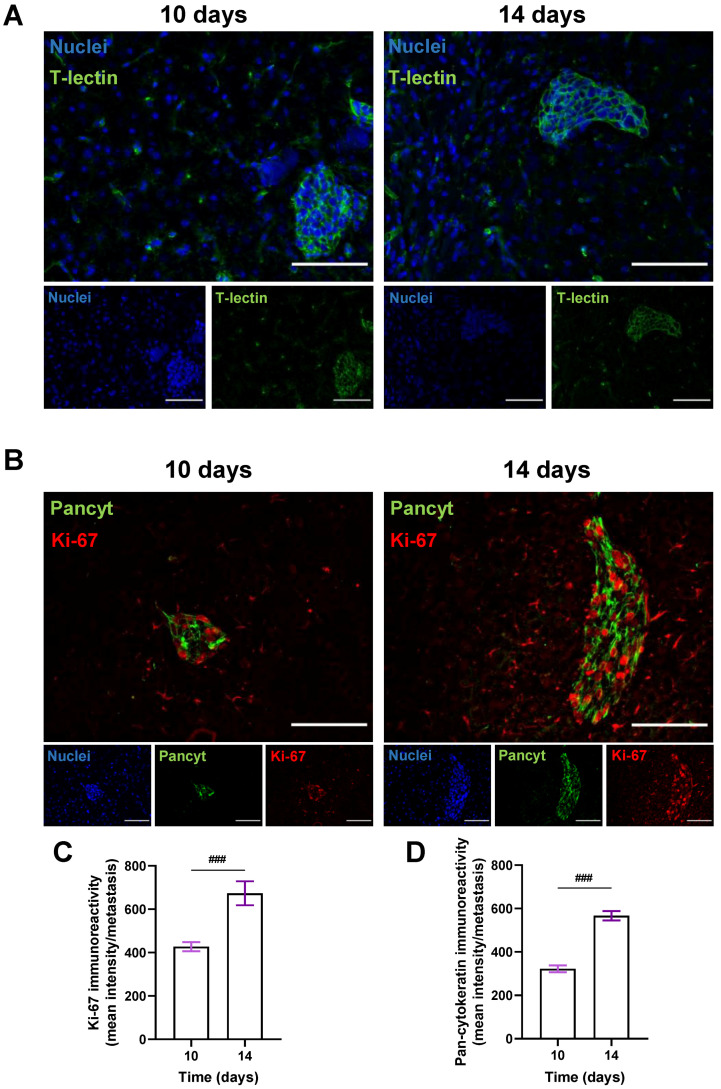
Characterization of the epithelial and proliferative features of breast cancer cells in brain metastases. 4T1 cells were injected in the common carotid artery of BALB/c mice and fluorescence/immunofluorescence analysis was performed in cranial hippocampal sections 10 and 14 days after. The epithelial marker tomato-lectin (T-lectin, green) fluorescence revealed T-lectin-positive brain metastases (**A**). Double labelling with a proliferative and an epithelial marker, Ki-67 (red) and pan-cytokeratin (Pancyt, green), respectively, showed Ki-67- and Pancyt-positive cells in metastases (**B**). Scale bar: 100 µm. Semi-quantitative analysis of Ki-67 and Pancyt showcased an increase in the mean intensity in a time-dependent manner (**C**,**D**). Hoechst dye 33342 was used as counterstaining for nuclei (blue). Data are given as mean ± SEM (*n* = 3; 10 fields/condition). A Kolmogorov–Smirnov test was used to evaluate the significant differences between different timepoints. Statistical significance is denoted as ### *p* < 0.001 between timepoints.

To better visualize metastatic lesions and understand the BCCs’ phenotype in BM, the epithelial markers T-lectin and Pancyt expression by malignant cells were inspected. To also discern if an increasing metastatic area over time resulted from a progressive proliferative capacity of BCCs, the expression of the proliferation marker Ki-67 was assessed in the cranial hippocampus 10 and 14 days after BCCs injection (Figure 4). An epithelial phenotype in BCBM was depicted by the expression of T-lectin (Figure 4A). A proliferative phenotype (Ki-67-positive BCCs in BM), along with a substantial Pancyt expression 10 days after the inoculation of 4T1 cells, was observed (Figure 4B). Notably, an increase in Ki-67 immunoreactivity was visible from 10 to 14 days (Figure 4C), reflecting metastatic cells’ proliferation during BCBM progression, in accordance with the enlargement of the metastases’ area from 10 to 14 days (Figure 2F). Furthermore, an increase in Pancyt immunoreactivity was also denoted throughout time (Figure 4D), emphasizing the manifestation of epithelial characteristics by tumour cells. The overall results illustrate an upregulation of epithelial markers in BCCs as metastases’ development progresses, resembling the original tissue features.

## 4. Discussion

Although improvements have been achieved over the years with in vitro models, namely by increasing their complexity with the use of 3D models or microfluidics [26], they are still incapable of fully recapitulating the complete in vivo setting, limiting the study of mechanisms related to the microenvironment. Therefore, there is a need for faithful animal models that accurately address all the molecular and cellular processes involved in BCBM formation. To closely address the mechanisms behind BM development and to have a minimal impact on the BBB and brain itself, it is imperative to adopt an ectopic approach such as the intracarotid injection route. In contrast to other experimental BM animal models, the intracarotid artery injection route presents a higher reproducibility while evading injection-induced traumatic injury and a compromised BBB [24,27]. Interestingly, the intracarotid injection of the murine mammary carcinoma 4T1 cell line, which is an aggressive, highly tumorigenic and invasive TN tumour model, promotes spontaneous metastases development in a pattern that is analogous to human mammary cancer [15]. Additionally, as opposed to other models whose growth and progression are not akin to human BC [15,28], 4T1 cells’ intracarotid injection produces a smaller variation of experimental results [18] and allows for the use of immunocompetent mice, since both the cells and host are from the same species [16]. Being previously applied in other studies [25,27,29], this model requires exceptional microsurgical skills to successfully direct BCCs to the brain, particularly the hippocampus, comprising a suitable strategy to study BCBM development [25,29]. The use of the intracarotid injection model still has some hurdles, as it only enables the recapitulation of the later steps of the metastatic cascade when compared with the fat-pad injection, which can recapitulate all of the metastization process. Importantly, the perfect model for a BM study may not be achievable, and while allowing for taking into consideration the physiology of the BC metastatic cascade, a fat-pad injection model would certainly lead to metastases developing primarily in various organs other than the brain, as others have already described [30], with a significant mortality rate before the development of brain metastases. On the other hand, the intracarotid injection model provides a unique platform to study the potential for biomarkers and assess the BBB permeation of new BBB therapeutic agents for the treatment of BM. Moreover, it is key for dissecting the several molecular events associated with tumour cells’ transmigration through the BBB endothelium and brain colonization, which is not so exclusively achieved with other injection routes.

The shortage of comprehensive studies disclosing both the hurdles and tricks of the method implementation as well as information about cellular and molecular events upon BCBM formation prompted us to go further and divulge the small but important technical skills of the procedure and characterize BCCs properties upon BCBM development. In fact, a mouse model of BCBM relying on the inoculation of murine mammary carcinoma TN 4T1 cells was previously used [25,29,31,32]. This model consisted of the inoculation of 1 × 10^6^ 4T1 cells in 200 µL of Ringer-HEPES in the CCA of 8–10-week-old female mice to direct the malignant cells to the brain and allow preferential BM formation [25,29]. This model was characterized by a high number of BM in coronal sections of the cranial hippocampus, with a lower and similar pattern of metastases’ formation in the cerebellum and in the lungs [25,29]. However, in our case, the intracarotid injection of the referred amount of 4T1 cells led to an extensive rate of death by an embolism. Strikingly, the animals presented a higher survival rate when the total cellular volume was not injected, suggesting that a change in the experimental procedure was imperative. As a result, we implemented several adaptations to the reported method, most notably the reduction in the cell amount to 0.2 × 10^6^ 4T1 cells, in line with other reports for TNBC cells’ injection in the carotid artery [27]. Moreover, as an improvement to our model and to better expose the surgical area and carotid artery, a cotton swab was placed under the animal’s neck, which was revealed to be essential to improve the injection itself. Also, in an attempt to reduce haemorrhaging in response to the surgery, at the point of injection, a haemostatic tampon was used to stop and prevent bleeding post-injection.

The histological analysis of the cranial hippocampus revealed the presence of metastases at 7 days as well as a gradual expansion in their number and area throughout time. Our data are in alignment with Lorger and colleagues, who observed an increased metastatic burden 10 days after the intracarotid injection of 4T1 cells. Of note is that the amount of cells injected by Lorger et al. (1 × 10^5^ cells) [33] is comparable to our model, where we injected 2 × 10^5^ cells. Moreover, they demonstrated that at 7 days post-injection, the cells were found in the extravascular area, therefore in the brain parenchyma [33], which is in line with our data, where from 7 days onwards, BC metastases in the brain parenchyma were observed. On the contrary, no significant metastases were detected in the cerebellum, which is consistent with previous studies [25]. To evaluate the metastatic progress in peripheral organs, the lungs, kidneys and livers were subjected to a histological analysis with HE staining, in which metastases were only observed in the lungs, with an increase in the tumour area throughout time. This observation was corroborated by Ki-67 staining, which revealed a time-dependent increase in Ki-67 expression in the lungs, with a marked expression at 14 days post-BCC-injection, which contrasts with the lack of expression in the liver and kidneys.

One could inquire about the brain blood supply after the CCA ligation. In fact, there are two carotid arteries (right and left) [34], and in this study, the procedure was performed in the right one. Additionally, we did not compromise the flux, as there was still circulatory flux through the left common carotid. Moreover, not only the internal carotid artery but also the vertebral artery is responsible for the brain blood supply. Once the blood covers the entirety of the brain, its supply is maintained via the circle of Willis, which gives rise to blood circulation reaching all of the brain (already being described as acting as a protection against ischemia upon vessel damage) [34]. Even though we performed the ligation of the CCA, we still observed lung metastases. This suggests that not all the cells are arrested in the capillaries within the brain, with a fraction continuing in the circulation and arresting in a pulmonary capillary bed to form lung metastases. However, it was previously reported that the extension of lung metastases was similar to that observed in the cerebellum, the least affected brain region [25]. Altogether, the injection of BCCs in the common carotid artery appears to be an in vivo model of the preferential formation of BM from BCCs.

To further characterize the present BCBM mouse model, we evaluated the proliferation profile of BCCs upon brain colonization by assessing the expression of the proliferation marker Ki-67 [35]. A marked increase in Ki-67-positive cells was observed along BCBM progression, showcasing a progressive proliferative phenotype of BCCs with the increase in the metastatic area over time. These results are aligned with the previous notion that high levels of Ki-67 expression in BC strongly correlate with a more tenacious proliferation, disease severity and, consequently, poor prognosis [29,36]. Together with Ki-67, Pancyt, an epithelial marker expressed by malignant cells, was evaluated and shown to increase with the metastatic stage of the tumour, being concomitant with previously obtained results in our laboratory [25,29]. This is reinforced by the expression pattern of the epithelial marker T-lectin in established BCBM throughout time—also in line with our previous studies [29]. Overall, our results illustrate an upregulation of epithelial markers in BCCs, allowing them to adapt and proliferate in the brain microenvironment during BCBM development [6]. With our work, it was possible to implement and characterize a reliable animal model of BCBM, essential for the study of BM formation, discovery of biomarkers and to ascertain the potential of therapeutic strategies directed at such pathology.

## 5. Conclusions

Overall, we were successful in developing a strong intracarotid animal model of BCBM formation that accurately mimics the in vivo setting of the process. Through the progression of metastases over time, we discovered that the hippocampus was a severely afflicted area of the brain, with no significant metastases observed in the cerebellum. Only the lungs demonstrated metastatic development among the peripheral organs. Furthermore, after colonization, BCCs’ metastatic properties were demonstrated by the acquisition of a proliferative ability (Ki-67) and the adoption of an epithelial phenotype (Pancyt and T-lectin). Later in time, the number of metastases in the brain stabilized, demonstrating the transition from the extravasation phase to brain colonization. Altogether, this work shortens the path to confront BCBM development in vivo through the implementation and characterization of a reliable animal model of BCBM development. Importantly, this research opens the door to its usage for preclinical studies, highlighting the therapeutical potential of this model. Its research applications are not restricted to the exploration of the pathophysiology and cellular mechanisms of BM formation, but also the prevention of BM formation or the treatment of already established BM. Moreover, this work paves the way to this model being used for preclinical studies that are essential to ascertain the potential of therapeutic strategies directed not only at BCBM but also at BM from other types of cancer that are prone to form BM, like lung cancer and melanoma.

## Figures and Tables

**Table 1:** Summary of the antibodies used in immunofluorescence analysis.

Marker	Primary Antibody	Secondary Antibody
Ki-67	Ki-67 (1:100) Thermo Fisher Scientific #PA5-19462, Rabbit Pc	Alexa Fluor^®^ 555 (1:500) Thermo Fisher Scientific, #A-21428, Goat anti-Rabbit
Pan-cytokeratin	Pan-Cytokeratin (1:100) Thermo Fisher Scientific #MA5-12231, Mouse Mc	Alexa Fluor^®^ 488 (1:500) ThermoFisher Scientific, #A-11001 Goat anti-Mouse

Mc, monoclonal; Pc, polyclonal.

## Data Availability

Not applicable.
